# Long-term psychosocial outcomes of low-dose CT screening: results of the UK Lung Cancer Screening randomised controlled trial

**DOI:** 10.1136/thoraxjnl-2016-208283

**Published:** 2016-07-28

**Authors:** Kate Brain, Kate J Lifford, Ben Carter, Olivia Burke, Fiona McRonald, Anand Devaraj, David M Hansell, David Baldwin, Stephen W Duffy, John K Field

**Affiliations:** 1 Cardiff University School of Medicine, Cardiff, UK; 2 Public Health England, Liverpool, UK; 3 Royal Brompton and Harefield NHS Foundation Trust, London, UK; 4 Department of Respiratory Medicine, Nottingham University Hospitals, Nottingham, UK; 5 Queen Mary University of London, London, UK; 6 University of Liverpool, Liverpool, UK

**Keywords:** Lung Cancer, Imaging/CT MRI etc, Psychology

## Abstract

**Background:**

The UK Lung Cancer Screening (UKLS) trial is a randomised pilot trial of low-dose CT (LDCT) screening for individuals at high risk of lung cancer. We assessed the long-term psychosocial impact on individuals participating in the UKLS trial.

**Methods:**

A random sample of individuals aged 50–75 years was contacted via primary care. High-risk individuals who completed T_0_ questionnaires (baseline) were randomised to LDCT screening (intervention) or usual care (no screening control). T_1_ questionnaires were sent 2 weeks after baseline scan results or control assignment. T_2_ questionnaires were sent up to 2 years after recruitment. Measures included cancer distress, anxiety, depression and decision satisfaction.

**Results:**

A total of 4037 high-risk individuals were randomised and they completed T_0_ questionnaires (n=2018 intervention, n=2019 control). Cancer distress was higher at T_1_ in intervention arm participants who received positive screening results (p≤0.001), but not at T_2_ (p=0.04). T_2_ anxiety (p≤0.001) and depression (p≤0.01) were higher in the control arm, but the absolute differences were small and not clinically relevant. At both time points, fewer control than screened participants were satisfied with their decision to participate in UKLS (p≤0.001). Regardless of trial allocation, cancer distress was higher in women (p≤0.01), participants aged ≤65 years (p≤0.001), current smokers (p≤0.001), those with lung cancer experience (p≤0.001) and those recruited from the Liverpool area (p≤0.001).

**Conclusion:**

Lung cancer screening using LDCT appears to have no clinically significant long-term psychosocial impact on high-risk participants. Strategies for engaging and supporting underserved groups are the key to implement routine lung cancer screening in the UK.

**Trial registration number:**

ISRCTN 78513845; results.

**Key messages**

**What is the key question?**

- What is the long-term psychosocial impact on high-risk individuals taking part in a UK randomised pilot trial of low-dose CT lung screening?

**What is the bottom line?**

- Low-dose CT screening appears to have no clinically significant psychosocial impact on high-risk participants in the long term, but subgroups including women, younger participants, smokers, those with experience of lung cancer and those living in deprived areas may require targeted information and support.

**Why read on?**

- The evidence will contribute to clinical and policy decisions regarding the successful and equitable implementation of low-dose CT lung screening for high-risk individuals in the UK.

## Introduction

Lung cancer is the leading cause of cancer-related mortality in the UK.^1^ Although improving, the 5-year survival rate for lung cancer in the UK is approximately 10% and lower than in other countries with comparable healthcare systems.^2^ This is partly due to patients presenting at an advanced disease stage, with over 65% of cases diagnosed at stage III or stage IV^3^ when treatment is usually palliative.

Effective early detection strategies are critical for enabling earlier diagnosis, curative treatment and better lung cancer prognosis. Lung cancer screening using low-dose CT (LDCT) in high-risk groups has demonstrated a 20% relative reduction in lung cancer mortality compared with chest X-ray in the US National Lung Screening Trial (NLST).^4^ However, false-positive rates of 20%–50% have been reported.^4^ ^5^ Policy decisions about whether to implement a new screening technology require evidence regarding psychosocial consequences that may influence successful application in routine practice.

Previous controlled trials have reported the psychosocial effects of LDCT lung cancer screening. The Danish Lung Cancer Screening Trial (DLCST)^6^ ^7^ and Dutch-Belgian NELSON trial^8^ found no differential effect of trial allocation on a range of psychosocial outcomes at 1 and 2 years follow-up, respectively. Temporary adverse effects of receiving abnormal results have been observed in high-risk participants randomised to LDCT screening.^9–11^ The NELSON trial reported poorer quality of life and increased anxiety and cancer distress at 2 months follow-up in recipients of an indeterminate scan result.^11^ However, these effects had diminished at 6 months follow-up.^8^ Recently, the NLST reported no significant differences between those receiving an abnormal and those receiving normal lung screening result in anxiety and health-related quality of life at 1 and 6 months follow-up.^12^

The UK Lung Cancer Screening (UKLS) pilot trial is the first to assess the feasibility, cost-effectiveness and psychosocial impact of lung cancer screening using a single LDCT screen versus no screening in a UK high-risk population.^13^ When considering the impact of lung cancer screening, it is important to understand the moderating role of participant characteristics that could present barriers to successful implementation. For example, smokers may perceive few benefits of screening^14^ ^15^ and harbour fatalistic and avoidant beliefs about lung cancer^16–18^ compared with former or non-smokers. Individuals from poorer backgrounds may be less enthusiastic about screening and face more barriers than those from affluent backgrounds.^19^ In earlier UKLS reports, trial participation was less likely in smokers, women, older age groups, those with higher levels of concern about lung cancer and those in lower socioeconomic groups.^20^ ^21^

We report the effects of UKLS trial participation on short-term and long-term psychosocial outcomes. The primary hypothesis was that intervention arm participants—in particular those with a positive (ie, abnormal) baseline scan result—would report higher short-term cancer distress compared with those in the control arm, but there would be no differential effects of trial arm or screening outcome on long-term distress. In secondary analyses of outcomes adjusted for a range of covariates, it was anticipated that subgroups including smokers and those from socioeconomically deprived areas would report adverse long-term outcomes regardless of trial allocation or result.

## Methods

### Participants and procedures

A random sample of 247 354 individuals aged 50–75 years residing in six recruitment centres at two sites (Liverpool, Knowsley and Sefton; Cambridgeshire, Peterborough and Bedfordshire) was sent trial information packs that included a questionnaire regarding lung cancer risk factors. From the questionnaire responders, 8729 patients were identified as having high risk of lung cancer (≥5% over 5 years) using the LLP_v2_ risk prediction model.^13^ Characteristics of trial non-participants are reported elsewhere.^20^ ^21^

Following completion of a further questionnaire to identify trial eligibility, those meeting the criteria were invited to attend their local recruitment centre in the Liverpool or Cambridge area. High-risk individuals who gave informed written consent were randomly allocated by simple computer pseudo-random number generation to the intervention (LDCT) or control arms in a 1:1 ratio.^13^

Participants completed a touchscreen questionnaire that included baseline psychosocial measures (T_0_). A second psychosocial questionnaire (T_1_) was sent approximately 2 weeks after receiving either a letter of assignment to the control group or a baseline CT scan result letter (intervention arm). T_2_ psychosocial questionnaires were sent in a single mailshot during January 2014.

### Measures

*Primary outcome: lung cancer distress* was measured using the six-item Cancer Worry Scale^22^ ^23^ anchored to thoughts and feelings about lung cancer during the past week. The scale had good internal consistency (α>0.81). Total score range was 6–24, with a score above 12.5 corresponding to a clinically significant threshold score on the General Health Questionnaire-28.^24^

*Secondary outcomes: anxiety and depression* were measured using the Hospital Anxiety and Depression Scale.^25^ Anxiety and depression subscales include seven items on a 0–3 scale, anchored to how participants felt in the last week (score range 0–21) and with good internal consistency (α>0.73). Scores of 0–7 are classified as ‘normal’, 8–10 as ‘mild’ anxiety or depression and 11–14 and 15–21 as ‘moderate’ and ‘severe’, respectively.^25^ *Decision satisfaction* was assessed with the six-item Satisfaction with Decision Scale.^26^ Scores were calculated and averaged so that possible scores ranged from 1 to 5 (α>0.94). Due to bi-modal distribution in the present study, a binary variable was created to reflect lower decision satisfaction (score <5, ‘not very satisfied’) and higher decision satisfaction (score=5, ‘very satisfied’).

*Demographic variables*: age and gender were obtained from medical records. Participants were identified as current, ex-smokers or never-smokers using self-reported smoking status included in the UKLS risk questionnaire. Socioeconomic deprivation was measured using Index of Multiple Deprivation (IMD) scores calculated from postcodes and ranked into standard deprivation quintiles (quintile 1=most deprived, quintile 5=least deprived). Educational attainment, marital group, ethnicity and experience of lung cancer (self and/or close others) were included in the T_0_ questionnaire.

#### Screening results

**Figure 1:**
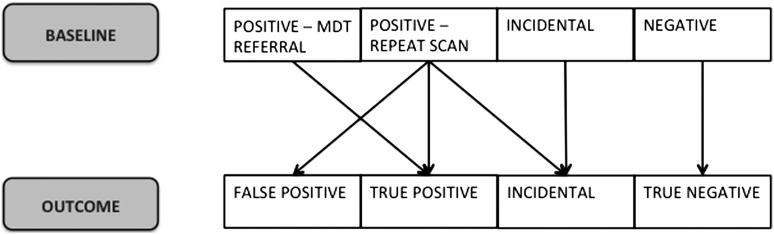
Screening results.

As shown in figure 1, at T_1_, we categorised baseline CT scan results into those that required a repeat scan in 3 or 12 months (positive for repeat scan) or referral to the multidisciplinary team (MDT) due to a major lung abnormality (positive for MDT referral), normal (negative) and significant incidental findings such as aortic aneurisms and pneumonia but with no findings suspicious for lung cancer (incidental finding). Categorising baseline CT scan results as ‘positive for repeat scan’ at T_1_ enabled comparison of the short-term responses of participants whose initial screening results were not unequivocally normal/negative. At T_2_, we distinguished screening outcome for participants with a positive baseline screening result who were free from lung cancer (false-positive) from those who did have lung cancer (true-positive) at long-term follow-up, those with normal results (true-negative) and those with significant abnormalities during baseline or repeat CT scan that were not lung cancer (incidental finding).

### Statistical analysis

Analyses were conducted using SPSS (V.20; SPSS, Chicago, Illinois, USA). Mean replacement imputation within domains was used and a complete case analysis was carried out. Individuals with missing data >35% within a domain were not included in the analysis. Attrition bias was assessed using χ^2^ and independent t tests. During primary analysis, psychosocial outcomes of trial allocation and screening results at T_1_ and T_2_ were assessed using analysis of covariance (ANCOVA) adjusted for T_0_ scores or χ^2^ tests. Due to non-normality and heterogeneity of regression slopes for T_1_ cancer distress scores, analyses of trial allocation effect on the primary outcome were conducted separately for log-transformed (log_n_) high and low cancer distress thresholds, that is, above or below 12.5.^24^ Sensitivity analyses were conducted to assess possible confounding effects of data timing issues and to adjust for missing follow-up data at T_1_ and T_2_ using an inverse probability weighting (IPW) approach.^27^ The p values ≤0.01 were used to denote statistical significance in the context of multiple testing. Following significant ANCOVA results, post-hoc pairwise comparisons of screening results were performed (p≤0.05).

A secondary linear mixed-effects risk prediction model was generated to evaluate the impact of trial allocation on the primary outcome of cancer distress at T_1_ and T_2_ in (1) univariable regression analyses adjusting for T_0_ distress scores and (2) multivariable regression analyses adjusting for T_0_ scores and all other main effects (gender, age group, smoking, deprivation quintile, education, ethnicity, marital group, lung cancer experience, trial site and time since attended recruitment centre). The model fitting process included mechanistically plausible confounders that exhibited independent associations in a forward-stepping method using the likelihood ratio statistic (p≤0.01), as part of an a priori statistical analysis plan. Since only intervention group participants received a CT scan, trial allocation group was fully nested with the result group.

## Results

### Trial participation

**Figure 2:**
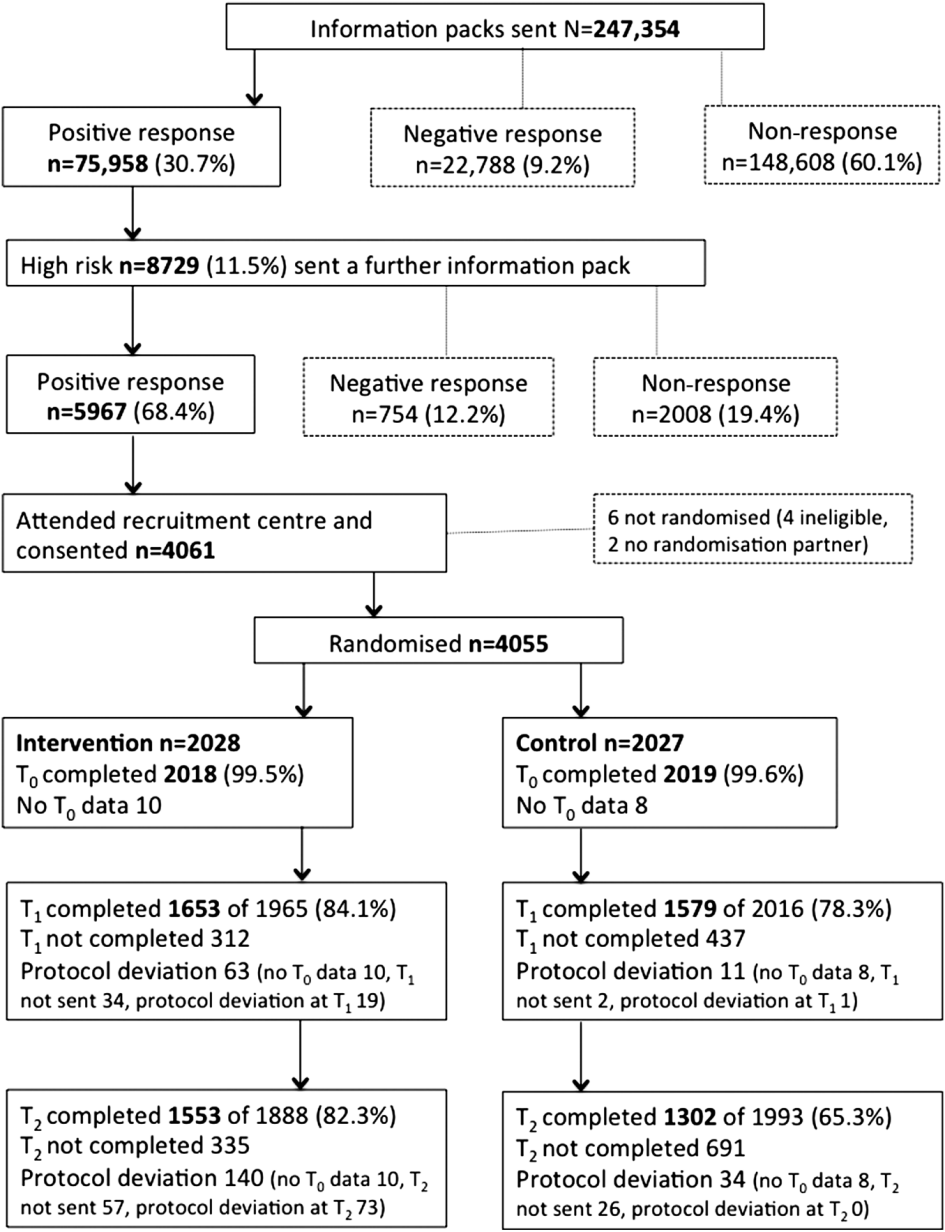
Trial flow chart.

In total, 4061 individuals (5.3% of 75 958 responders to the risk questionnaire; 46.5% of all high-risk positive responders) attended the recruitment clinic and were consented (see figure 2). Of these, 4037 trial participants completed T_0_ questionnaires and were randomised (n=2018 intervention, n=2019 control), of whom 3232 completed T_1_ questionnaires (n=1653/84% intervention, n=1579/78% control). At T_2_, 2855 participants completed questionnaires (n=1553/82% intervention, n=1302/65% control). The mean time of T_2_ questionnaire completion was 16 months after attending the recruitment centre (range 10–29 months).

### Factors associated with study attrition

As shown in tables 1 and 2, intervention participants were statistically significantly more likely than those in the control arm to complete T_1_ and T_2_. Questionnaire completers at T_1_ and T_2_ were significantly more likely to be older, male, married or cohabiting, more educated, former smokers, resident in the Cambridge area and those who have had no experience of lung cancer. Due to missing data for educational level, results for this variable should be interpreted with caution. A significantly greater proportion of questionnaire completers were in the highest IMD quintile at T_1_ and T_2_. T_1_ and T_2_ completers reported significantly lower baseline (T_0_) scores on all psychosocial measures, although the absolute differences were very small and scores were in the low range overall. Differences in decision satisfaction were not statistically significant at either follow-up.

**Table 1:** Attrition at short-term follow-up: comparison of T_1_ questionnaire completers and non-completers

	T_1_ completers (n=3232)* n (%) or mean (SD)	T_1_ non-completers (n=749)* n (%) or mean (SD)	Test statistic (p value)
Trial allocation			
Intervention	1653 (51)	312 (42)	<0.001
Control	1579 (49)	437 (58)	
Site			
Liverpool recruitment centre	1585 (49)	420 (56)	<0.001
Cambridge recruitment centre	1647 (51)	329 (44)	
Age (years)	67.73 (3.98)	67.28 (4.46)	<0.01
Gender			
Male	2446 (76)	533 (71)	<0.01
Female	786 (24)	216 (29)	
Education†			
Up to GCSE/O level	1007 (43)	334 (57)	<0.001
Beyond GCSE/O level	1310 (57)	250 (43)	
Ethnicity			
White	3190 (99)	739 (99)	‡
Non-white	26 (1)	10 (1)	
Marital group			
Married/cohabiting	2410 (75)	508 (68)	<0.001
Not married/cohabiting	814 (25)	240 (32)	
IMD			
Quintile 1 (most deprived)	843 (26)	222 (30)	<0.001
Quintile 2	367 (11)	109 (15)	
Quintile 3	574 (18)	135 (18)	
Quintile 4	578 (18)	141 (19)	
Quintile 5 (least deprived)	870 (27)	142 (19)	
Smoking status			
Current smoker	1194 (37)	338 (45)	<0.001
Ex-smoker	2037 (63)	410 (55)	
Never smoker	1 (<1)	1 (<1)	‡
Experience of lung cancer (T_0_)			
No	1870 (58)	396 (53)	<0.01
Yes	1355 (42)	352 (47)	
Cancer distress (T_0_)§	2.16 (0.28) *8.65*	2.21 (0.31) *9.16*	<0.001
Anxiety (T_0_)§	1.52 (0.71) *3.59*	1.65 (0.71) *4.20*	<0.001
Depression (T_0_)§	1.27 (0.67) *2.56*	1.37 (0.66) *2.94*	<0.001
Decision satisfaction (T_0_)			
Not very satisfied	1916 (59)	428 (57)	0.32
Very satisfied	1308 (41)	317 (43)	

*Ns vary in each cell due to missing data. Percentages were calculated based on available data.

†A substantial amount of data were missing or uninformative for education.

‡Data were excluded from analysis due to limited variation.

§Log_n_ scores are provided in normal text and original scale scores are provided in italics (analyses performed using log_n_ scores).

GCSE, General Certificate of Secondary Education; IMD, Index of Multiple Deprivation.

**Table 2:** Attrition at long-term follow-up: comparison of T_2_ completers and non-completers

	T_2_ completers (n=2855)* n (%) or mean (SD)	T_2_ non-completers (n=1026)* n (%) or mean (SD)	Test statistic (p value)
Trial allocation			
Intervention	1553 (54)	335 (33)	<0.001
Control	1302 (46)	691 (67)	
Site			
Liverpool recruitment centre	1336 (47)	622 (61)	<0.001
Cambridge recruitment centre	1519 (53)	404 (39)	
Age (years)	67.79 (3.98)	67.19 (4.31)	<0.001
Gender			
Male	2166 (76)	736 (72)	<0.01
Female	689 (24)	290 (28)	
Education†			
Up to GCSE/O level	940 (44)	367 (53)	<0.001
Beyond GCSE/O level	1196 (56)	330 (47)	
Ethnicity			
White	2825 (99)	1004 (99)	‡
Non-white	21 (1)	15 (1)	
Marital group			
Married/cohabiting	2174 (76)	672 (66)	<0.001
Not married/cohabiting	675 (24)	352 (34)	
IMD			
Quintile 1 (most deprived)	668 (23)	373 (36)	<0.001
Quintile 2	338 (12)	126 (12)	
Quintile 3	523 (18)	170 (17)	
Quintile 4	545 (19)	157 (15)	
Quintile 5 (least deprived)	781 (27)	200 (19)	
Smoking status			
Current smoker	1009 (35)	490 (48)	<0.001
Ex-smoker	1845 (65)	536 (52)	
Never smoker	1 (<1)	0 (0)	‡
Experience of lung cancer (T_0_)			
No	1683 (59)	522 (51)	<0.001
Yes	1166 (41)	503 (49)	
Cancer distress (T_0_)§	2.16 (0.28) *8.64*	2.20 (0.32) *9.06*	<0.001
Anxiety (T_0_)§	1.51 (0.71) *3.52*	1.64 (0.71) *4.17*	<0.001
Depression (T_0_)§	1.25 (0.66) *2.51*	1.38 (0.68) *2.96*	<0.001
Decision satisfaction (T_0_)			
Not very satisfied	1668 (59)	612 (60)	<0.47
Very satisfied	1179 (41)	410 (40)	

*Ns vary in each cell due to missing data. Percentages were calculated based on available data.

†A substantial amount of data were missing or uninformative for education.

‡Data were excluded from analysis due to limited variation.

§Log_n_ scores are provided in normal text and original scale scores are provided in italics (analyses performed using log_n_ scores).

GCSE, General Certificate of Secondary Education; IMD, Index of Multiple Deprivation.

### Baseline sample characteristics

Most trial participants were male, white, married or cohabiting, former smokers, with an average age of 68 years (see table 3). Of those for whom educational data were available, just over half the participants were educated beyond 16 years. There was a spread of deprivation levels within the sample, with approximately one-quarter in the highest quintile and one-quarter in the lowest quintile. T_0_ cancer distress, anxiety and depression scores were equivalent across trial arms. Approximately 40% were very satisfied with their decision to take part. The baseline characteristics of participants who were included in T_1_ and T_2_ analyses are presented in additional online supplementary tables SI and SII.

**Table 3:** Baseline psychosocial sample characteristics by trial allocation

	Intervention (n=2018)* n (%) or mean (SD)	Control (n=2019)* n (%) or mean (SD)
Site		
Liverpool recruitment centre	1003 (50)	1016 (50)
Cambridgeshire recruitment centre	1002 (50)	1016 (50)
Age (years)	67.72 (4.04)	67.59 (4.13)
Gender		
Male	1520 (75)	1500 (74)
Female	498 (25)	519 (26)
Education†		
Up to GCSE/O level or equivalent	678 (46)	683 (46)
Beyond GCSE/O level or equivalent	788 (54)	791 (54)
Ethnicity		
White	1992 (99)	1992 (99)
Non-white	18 (1)	19 (1)
Marital group		
Married/cohabiting	1483 (74)	1471 (73)
Not married/cohabiting	528 (26)	545 (27)
IMD		
Quintile 1 (most deprived)	545 (27)	533 (26)
Quintile 2	243 (12)	242 (12)
Quintile 3	358 (18)	361 (18)
Quintile 4	353 (18)	376 (19)
Quintile 5 (least deprived)	519 (26)	507 (25)
Smoking status		
Current smoker	772 (38)	787 (39)
Ex-smoker	1244 (62)	1232 (61)
Never smoker	2 (<1)	0 (0)
Experience of lung cancer (T_0_)		
No	1168 (58)	1126 (56)
Yes	846 (42)	889 (44)
Cancer distress (T_0_)‡	2.17 (0.29) *8.75*	2.17 (0.29) *8.74*
Anxiety (T_0_)‡	1.55 (0.71) *3.72*	1.54 (0.71) *3.67*
Depression (T_0_)‡	1.30 (0.68) *2.66*	1.28 (0.67) *2.61*
Decision satisfaction (T_0_)		
Not very satisfied	1228 (61)	1158 (58)
Very satisfied	786 (39)	853 (42)

*Ns vary in each cell due to missing data. Percentages were calculated based on available data.

†A substantial amount of data were missing or uninformative for education.

‡Log_n_ scores are provided in normal text and original scale scores are provided in italics (analyses performed using log_n_ scores).

GCSE, General Certificate of Secondary Education; IMD, Index of Multiple Deprivation.

10.1136/thoraxjnl-2016-208283.supp1 Supplementary table

10.1136/thoraxjnl-2016-208283.supp2 Supplementary table

### Primary analyses

#### Short-term (T_1_) outcomes

As shown in table 4, different effects of trial allocation on T_1_ cancer distress were found in participants who scored above and below the T_0_ distress threshold (12.5). For those with low T_0_ distress (n=2896/3225, 90%), T_1_ distress scores were significantly higher in the intervention arm, though not to clinical levels and with a very small effect size (log_n_ (Int-Con)=0.03, 95% CI 0.02 to 0.05). For those with high T_0_ distress (n=326/3225, 10%), the effect of trial allocation on T_1_ cancer distress was not significant: mean levels of cancer distress remained high and bordered on clinical levels regardless of trial allocation (153/326=47%) (log_n_ (Int-Con)=−0.04, 95% CI −0.09 to 0.01).

**Table 4:** Summary data for short-term psychosocial outcomes (T_1_) by trial arm and screening result

Outcome (T_1_)	Intervention (n=1653)	Control (n=1579)	Intervention arm (n=1653) by screening result†			
			Negative (n=763)	Incidental finding (n=41)	Positive—repeat scan (n=788)	Positive—MDT referral (n=48)
Cancer distress‡ M (95% CI)	Low T_0_ scorers 2.14 (2.13 to 2.16)** 8.54 (8.44 to 8.64)	Low T_0_ scorers 2.11 (2.10 to 2.12) *8.26 (8.16 to 8.36)*	2.12 (2.10 to 2.13) *8.32 (8.18 to 8.45)*	2.15 (2.08 to 2.22) *8.56 (7.97 to 9.19)*	2.23 (2.22 to 2.25)** 9.34 (9.19 to 9.49)	2.47 (2.41 to 2.54)** 11.88 (11.10 to 12.72)
	High T_0_ scorers 2.50 (2.46 to 2.53) *12.14* *(11.73 to 12.55)*	High T_0_ scorers 2.53 (2.50 to 2.57) *12.61* *(12.15 to 13.09)*				
Anxiety‡ M (95% CI)	1.54 (1.51 to 1.57) *3.67* *(3.54 to 3.80)*	1.56 (1.53 to 1.59) *3.78* *(3.64 to 3.92)*	1.51 (1.47 to 1.55) *3.54* *(3.35 to 3.73)*	1.50 (1.32 to 1.68) *3.49* *(2.75 to 4.39)*	1.56 (1.52 to 1.60) *3.76* *(3.57 to 3.96)*	1.87 (1.70 to 2.04)** 5.49 *(4.48 to 6.67)*
Depression‡ M (95% CI)	1.26 (1.23 to 1.29) *2.53* *(2.42 to 2.63)*	1.34 (1.31 to 1.37)** 2.81 *(2.70 to 2.92)*	1.27 (1.23 to 1.31) *2.55* *(2.41 to 2.70)*	1.20 (1.02 to 1.38) *2.31* *(1.76 to 2.97)*	1.26 (1.22 to 1.30) *2.51* *(2.37 to 2.66)*	1.40 (1.24 to 1.56) *3.05* *(2.44 to 3.78)*
Decision satisfaction (n, %) Not very satisfied Very satisfied	875 (58) 624 (42)	953 (66)** 498 (34)	378 (54) 324 (46)	22 (56) 17 (44)	450 (64)** 255 (36)	18 (43) 24 (57)

*p≤0.01, **p≤0.001.

Numbers vary in each cell due to missing data.

†n=13 excluded at T_1_ due to discrepancies in the classification of baseline CT scan results.

‡Log_n_ scores are provided in normal text and original scale scores are provided in italics (analyses performed using log_n_ scores). Estimated marginal means are presented. Higher scores denote higher levels of the relevant outcome.

The effect of trial allocation on T_1_ general anxiety was not statistically significant (log_n_ (Int-Con)=−0.02, 95% CI −0.06 to 0.02). Higher log_n_ depression scores were found in the control group, but the absolute difference was very small (log_n_ (Int-Con)=−0.08, 95% CI −0.12 to −0.04). When converted back to the original scale (0–21 range), depression scores were within the normal range for both groups. At T_1_, a significantly greater proportion of control arm participants were not very satisfied with their decision to take part (66%) compared with the intervention arm (58%) (p≤0.001). Sensitivity analyses to assess the impact of data timing showed no change in statistical significance levels; therefore, further exclusions were not made. Further adjustment for missing data using IPW indicated no change in the direction, magnitude or significance of the difference in cancer distress between intervention and control arms for either low or high scorers.

Intervention participants who were positive for MDT referral at T_1_ reported statistically significantly higher T_1_ cancer distress which approached clinical thresholds (p≤0.001) compared with those with negative results (p≤0.001), incidental findings (p≤0.001) and those positive for a repeat scan (p≤0.001). Participants who required a repeat scan at T_1_ reported significantly higher T_1_ cancer distress than those with negative results (p≤0.001). Statistically significantly greater anxiety was found in participants referred to MDT (p≤0.001) compared with those receiving negative results (p≤0.001), incidental findings (p=0.02) or positive for repeat scan (p=0.003), although scores were in the low/normal range. The difference in anxiety between those positive for MDT referral and those with incidental findings disappeared when sensitivity analysis accounted for test timing issues. Differences in depression scores were not statistically significant for any screening result group (p=0.35). Intervention participants who were positive for a repeat scan were the least satisfied with their decision (p≤0.001).

#### Long-term (T_2_) outcomes

The effect of trial allocation on T_2_ cancer distress was not statistically significant in individuals with low (log_n_ (Int-Con)=0.01, 95% CI −0.01 to 0.02) or high (log_n_ (Int-Con)=−0.02, 95% CI −0.09 to 0.05) T_0_ distress (see table 5). Although the control group reported significantly higher T_2_ log_n_ anxiety (log_n_ (Int-Con)=−0.08, 95% CI −0.12 to −0.03) and depression (log_n_ (Int-Con)=−0.06, 95% CI −0.10 to −0.02), the absolute differences between trial arms were small and not clinically significant. When converted to raw scores, all three measures for both trial arms were within the normal range. Control participants (74%) were significantly less likely than intervention participants (60%) to be very satisfied with their decision to take part (p≤0.001).

**Table 5:** Summary data for long-term psychosocial outcomes (T_2_) by trial arm and screening outcome

Outcome (T_2_)	Intervention (n=1553)	Control (n=1302)	Intervention arm (n=1553) by screening outcome†			
			True-negative (n=740)	Incidental finding (n=78)	False-positive (n=445)	True-positive (n=23)
Cancer distress‡ M (95% CI)	Low T_0_ scorers 2.10 (2.09 to 2.11) *8.15* *(8.05 to 8.25)*	Low T_0_ scorers 2.09 (2.08 to 2.10) *8.10* *(7.99 to 8.25)*	2.11 (2.09 to 2.12) *8.22* *(8.09 to 8.36)*	2.14 (2.09 to 2.19) *8.48* *(8.06 to 8.93)*	2.14 (2.12 to 2.16) *8.51* *(8.33 to 8.70)*	2.20 (2.10 to 2.30) *9.01* *(8.16 to 9.96)*
	High T_0_ scorers 2.44 (2.39 to 2.48) *11.43* *(10.93 to 11.95)*	High T_0_ scorers 2.46 (2.41 to 2.51) *11.69* *(11.11 to 12.30)*				
Anxiety‡ M (95% CI)	1.54 (1.51 to 1.57) *3.66* *(3.52 to 3.80)*	1.61 (1.58 to 1.65)** 4.02 *(3.86 to 4.19)*	1.57 (1.53 to 1.62) *3.82* *(3.61 to 4.03)*	1.45 (1.32 to 1.59) *3.28* *(2.74 to 3.89)*	1.52 (1.47 to 1.58) *3.59* *(3.34 to 3.85)*	1.37 (1.13 to 1.62) *2.94* *(2.08 to 4.03)*
Depression‡ M (95% CI)	1.33 (1.30 to 1.36) *2.77* *(2.67 to 2.89)*	1.39 (1.36 to 1.42)* 3.01 *(2.89 to 3.14)*	1.34 (1.30 to 1.39) *2.84* *(2.68 to 3.00)*	1.22 (1.09 to 1.35) *2.38* *(1.97 to 2.85)*	1.38 (1.33 to 1.44) *2.98* *(2.78 to 3.20)*	1.26 (1.02 to 1.49) *2.52* *(1.79 to 3.44)*
Decision satisfaction (n, %) Not very satisfied Very satisfied	855 (60) 567 (40)	883 (74)** 306 (26)	411 (61) 267 (39)	39 (55) 32 (45)	239 (59) 169 (41)	6 (29)§ 15 (71)

*p≤0.01, **p≤0.001.

Ns vary in each cell due to missing data.

†n=267 were excluded at T_2_ if they had no baseline scan, had a MDT referral but no lung cancer diagnosed, were awaiting scan or results, or had results that were not classified.

‡Log_n_ scores are provided in normal text and original scale scores are provided in italics (analyses performed using log_n_ scores). Estimated marginal means are presented. Higher scores denote higher levels of the relevant outcome.

§Test statistics not calculated due to low variation.

Differences between screening outcome groups in T_2_ cancer distress (p=0.04), anxiety (p=0.12), depression (p=0.11) and decision satisfaction (p=0.03) were not statistically significant. The raw scores of all psychosocial variables in all screening outcome groups were within the normal range and not clinically relevant. Sensitivity analyses using IPW to assess the impact of missing data indicated no change in the direction, magnitude or significance of the difference in cancer distress between intervention and control arms, either for low or high baseline scorers.

### Secondary analyses

In univariable analyses, the mean difference in log_n_ cancer distress scores between the intervention and control arms was not statistically significant after adjustment for T_0_ cancer distress and independent effect modifiers (see table 6). This equated to a relative increase of 2% for intervention arm participants when distress scores were back-transformed (95% CI 12% to 17%). Cancer distress scores were statistically significantly higher in women (p≤0.001), younger participants (≤65 years and 66–70 years) compared with those aged over 70 (p≤0.001), current smokers compared with ex-smokers (p≤0.001), lower socioeconomic groups (Q1-Q2 vs Q5 p≤0.001, Q3 vs Q5 p≤0.01), single or divorced compared with married individuals (p≤0.001), those with experience of lung cancer (p≤0.001) and participants recruited from the Liverpool area (p≤0.001). Education, ethnicity and time since recruitment were not significantly independently associated with cancer distress. Intervention participants who needed a repeat scan or MDT referral reported higher cancer distress than those who received a negative result (p≤0.001).

**Table 6:** Univariable and multivariable analyses of trial allocation effects on cancer distress over T_1_ and T_2_

Cancer distress effect modifiers	Log_n_ difference in cancer distress		
	Estimate (unadjusted), 95% CI, p value	Estimate (adjusted)*, 95% CI, p value
Trial allocation (Intervention–Control)	0.02 (−0.13 to 0.17) 0.42	0.03 (−0.20 to 0.26) 0.39
Gender (Female–Male)	0.04 (0.03 to 0.06) ≤0.001	0.02 (0.01 to 0.04) ≤0.01
Age group		
Up to 65–over 70	0.06 (0.05 to 0.08) ≤0.001	0.05 (0.03 to 0.07) ≤0.001
66 to 70–over 70	0.04 (0.02 to 0.05) ≤0.001	0.02 (0.02 to 0.04) 0.02
Smoking		
Ex-smoker–Current smoker	−0.08 (−0.06 to −0.09) ≤0.001	−0.06 (−0.05 to −0.08) ≤0.001
Never smoker†–Current smoker	†	†
IMD		
Q1 (most deprived)–Q5 (least deprived)	0.09 (0.07 to 0.10) ≤0.001	0.02 (−0.01 to 0.04) 0.13
Q2–Q5	0.05 (0.03 to 0.07) ≤0.001	0.01 (−0.02 to 0.03) 0.66
Q3–Q5	0.03 (0.01 to 0.04) ≤0.01	−0.00 (−0.02 to 0.02) 0.89
Q4–Q5	0.02 (0.00 to 0.04) 0.029	0.01 (−0.01 to 0.03) 0.59
Education (Up to GCSE/O level–beyond GCSE/O level)	0.01 (0.00 to 0.03) 0.05	–
Ethnicity (White–non-white)	0.01 (−0.06 to 0.08) 0.79	–
Marital group		
Married–Single	−0.04 (−0.02 to –0.07) ≤0.001	–
Married–Cohabiting	0.01 (−0.02 to 0.04) 0.41	–
Married–Widowed	−0.03 (−0.00 to –0.05) 0.03	–
Married–Divorced	−0.05 (−0.03 to –0.07) ≤0.001	–
Experience of lung cancer (Yes–No)	0.05 (0.03 to 0.06) ≤0.001	0.03 (0.02 to 0.05) ≤0.001
Site (Liverpool–Cambridge)	0.07 (0.06 to 0.08) ≤0.001	0.06 (0.04 to 0.07) ≤0.001
Time since attended recruitment centre		
3 to 6 months–<3 months	−0.00 (−0.03 to 0.03) 1.00	−0.03 (0.00 to −0.05) 0.09
6 to 12 months–<3 months	−0.05 (−0.09 to −0.01) 0.03	−0.06 (−0.11 to −0.01) 0.03
>12 months–<3 months	−0.03 (−0.12 to 0.06) 0.09	−0.05 (−0.00 to −0.10) 0.05
Screening result		
Positive (repeat scan)–Negative	0.09 (0.07 to 0.11) ≤0.001	–
Positive (referral)–Negative	0.20 (0.15 to 0.24) ≤0.001	–
Incidental–Negative	0.02 (−0.02 to 0.07) 0.33	–

Data are presented for participants with low T_0_ cancer distress (n=2896/3225, 90%). Base model adjustment factors included: T_0_ cancer distress, trial allocation, gender, age, smoking, deprivation, education, ethnicity, marital group, time since recruitment, recruitment site and experience of lung cancer.

*Adjusted for T_0_ cancer distress, trial allocation, gender, age, smoking, deprivation, time since recruitment, recruitment site and experience of lung cancer.

†Data were excluded due to limited variation.

GCSE, General Certificate of Secondary Education; IMD, Index of Multiple Deprivation.

In multivariable analyses adjusting for covariates, the impact of trial allocation on cancer distress was not statistically significant. Higher cancer distress was statistically significantly associated with female gender (p≤0.01), younger age group (≤65 vs over 70 years) (p≤0.001), current smoking status (p≤0.001), lung cancer experience (p≤0.001) and Liverpool recruitment site (p≤0.001).

Sensitivity analyses to assess the impact of T_0_ cancer distress level indicated a similar pattern of results for participants with high T_0_ cancer distress levels.

## Discussion

The present study is the first to report the long-term psychosocial impact of LDCT screening in a UK high-risk population. Transient negative consequences were observed in individuals allocated to LDCT screening and in those who received unfavourable screening results, but these differences were neither sustained over time nor clinically significant. However, a profile of risk factors emerged for adverse consequences of participating in lung cancer screening.

The overall findings confirm evidence from non-UK controlled trials that allocation to LDCT screening does not appear to produce long-term anxiety or other adverse effects that could potentially deter high-risk individuals from future adherence to lung screening. In studies with repeated screening such as NLST, the majority of participants returned for further screenings.^4^ NELSON^8^ and PLCO^9^ observed minimal long-term psychosocial effects of LDCT screening. Although UKLS participants who were assigned to the control group reported slightly higher long-term anxiety and depression, the absolute differences were very small and not clinically significant. This finding may reflect unscreened participants' disappointment or frustration at having been identified as high risk but denied the opportunity to gain reassurance from screening, supported by the finding that a greater proportion were less satisfied with their decision to participate compared with the intervention arm. Similarly, the DLCST reported negative consequences in the unscreened control arm at 1^6^ and 2 years.^7^

The short-term impact of UKLS participation largely reflected temporary adverse effects of positive screening results, which disappeared by 2 years follow-up. This finding supports previous LDCT trials that indicate long-term resolution of adverse screening effects.^8^ ^9^ ^11^ Unsurprisingly, participants who were referred due to a suspected major lung abnormality reported higher short-term cancer distress than any other screening result group, with levels close to threshold scores. Those who required a repeat scan reported higher distress than those receiving an immediate ‘all-clear’ result. Higher short-term anxiety was observed in participants with a suspected major lung abnormality, but scores were within the normal range and they were also more at ease with their decision to take part in the trial. The latter finding has been reported in other screening evaluation studies, suggesting decision consolidation and the perception that further diagnostic tests have been carried out thoroughly and for personal benefit.^28^

Individual difference variables that predicted higher levels of cancer distress over time, irrespective of trial allocation, included female gender and younger age (under 65 years). Women may feel more worried and less prepared for the prospect of lung cancer screening than men, possibly due to exposure to female cancer screening programmes and perceptions of lung cancer as a traditionally male disease. Smokers and those who had been exposed to lung cancer in their social networks were also more concerned about lung cancer. Perceived stigma and fatalism surrounding a lung cancer diagnosis are important issues, especially for smokers.^14^ ^16^ ^17^ This may be partly due to adverse vicarious experiences (ie, witnessing poor outcomes in family and friends with lung cancer) that influence the formation of negative beliefs and attitudes towards lung cancer screening. The links between smoking, socioeconomic deprivation and lung cancer incidence and mortality are well known^29^ ^30^ and psychosocial outcomes were indeed poorer in those recruited from the Liverpool area, which is known to have high levels of deprivation and high incidence of lung cancer.^31^ Supportive interventions to improve the quality of information and care and minimise potential distress in vulnerable groups, should be implemented alongside routine LDCT lung screening. In addition, individuals with high pre-existing cancer distress could be identified for psychosocial support.^24^

While the overall trend towards minimal psychosocial consequences of UKLS is encouraging, we acknowledge the possibility that sample selection bias may limit external validity. High-risk individuals who were older, female, smokers, from a lower socioeconomic group or more concerned about lung cancer were less likely to participate^21^ and continued to be under-represented as the trial progressed. These findings are consistent with barriers to uptake that have been reported in previous lung screening trials.^14^ ^16^ ^17^ ^32^ The current findings suggest that women may initially be less aware or convinced of the need for lung screening, compared with breast and cervical cancers where there are well-established screening programmes. In the DLCST, trial participation was similarly associated with deprivation and age, but in contrast to our findings men were under-represented in the DLCST.^33^ Further in-depth qualitative research is required to understand the perceptions and attitudes of men and women who are at high risk and eligible for lung cancer screening.

We also recognise that sample bias may have led to potential type II error in detecting long-term effects of LDCT screening, due to the relatively small number of participants with true-positive or significant incidental results. Gareen *et al*^12^ reported poorer health-related quality of life and higher state anxiety in NLST participants who received a lung cancer diagnosis. Some participants were excluded from screening outcomes analyses, for example, those referred but in whom lung cancer was not diagnosed. Long-term psychosocial effects in these individuals are unknown. Interestingly, there was no observed difference in distress between participants who had incidental non-lung cancer findings compared with those with negative results. However, the number of significant incidental findings may increase in routine practice compared with a trial setting. The potential for distress caused by unfavourable or unexpected findings of lung screening should not be ruled out. We acknowledge that, in future, the psychosocial consequences of lung cancer screening will need to be examined in the context of a health service (if implemented) and that the single-screen design of the UKLS trial may limit the generalisability of our findings to the context of enrolment in a routine screening programme.

LDCT lung screening is currently recommended in the US, but policy decisions in Europe await pooled data from European trials.^34^ There is an ethical imperative to promote informed participation in lung cancer screening prior to its routine implementation in the UK. Public and patient facing information materials should aim to prepare people for informed participation by increasing understanding of the purpose, benefits and risks of lung screening, including the possibility of receiving abnormal results and strategies for coping with associated short-term distress. Interventions that target smokers may improve informed participation in lung screening and provide a platform from which to engage smokers in smoking cessation services.

The present results must be interpreted alongside evidence of feasibility, cost-effectiveness and reach to determine whether LDCT lung cancer screening should become programmatic in the UK. Strategies for engaging and supporting high risk, harder to reach groups should also be trialled if lung cancer screening is to be successfully and equitably rolled out in the community setting.
