# Supplementary material for: Long-term psychosocial outcomes of low-dose CT screening: results of the UK Lung Cancer Screening randomised controlled trial
Source: Thorax. 2016 Jul 28;71(11):996–1005. doi: 10.1136/thoraxjnl-2016-208283 (PMC5099188; doi:10.1136/thoraxjnl-2016-208283)
Supplement: Supplementary table [file thoraxjnl-2016-208283supp_table1.pdf]

Supplementary Table I. T_1 sample baseline characteristics by trial allocation

			Intervention (n=1653)‡ n (%) or mean (SD)	Control (n=1579)‡ n (%) or mean (SD)	Test statistic (p value)
**Site**	Liverpool		819 (50)	766 (49)	p=0.56
	Cambridge		834 (51)	813 (52)	
**Age (years)**			67.71 (3.94)	67.75 (4.02)	p=0.77
**Gender**	Male		1253 (76)	1193 (76)	p=0.87
	Female		400 (24)	386 (24)	
**Education¶**	Up to GCSE/O level		516 (44)	491 (43)	p=0.53
	Beyond GCSE/O level		654 (56)	656 (57)	
**Ethnicity**	White		1632 (99)	1558 (99)	^
	Non-white		13 (1)	13 (1)	
**Marital group**	Married /cohabiting		1239 (75)	1171 (74)	p=0.57
	Not married/cohabiting		409 (25)	405 (26)	
**IMD**	Quintile 1 (most deprived)		447 (27)	396 (25)	p=0.51
	Quintile 2		193 (12)	174 (11)	
	Quintile 3		292 (18)	282 (18)	
	Quintile 4		280 (17)	298 (19)	
	Quintile 5 (least deprived)		441 (27)	429 (27)	
**Smoking status**	Current smoker		621 (38)	573 (36)	p=0.44
	Ex-smoker		1031 (62)	1006 (64)	
	Never smoker		1 (<1)	0 (0)	^
**Experience of lung cancer (T_0)**		No	974 (59)	896 (57)	p=0.20
		Yes	675 (41)	680 (43)	
**Cancer distress (T_0)⁺**			2.16 (0.28) *8.71*	2.15 (0.28) *8.60*	p=0.21
**Anxiety (T_0)⁺**			1.53 (0.71) *3.63*	1.51 (0.71) *3.55*	p=0.46
**Depression (T_0)⁺**			1.28 (0.68) *2.58*	1.26 (0.67) *2.54*	p=0.60
**Decision satisfaction (T_0)**	Not very satisfied		1000 (61)	916 (58)	p=0.18
	Very satisfied		651 (39)	657 (42)	

‡ Ns vary in each cell due to missing data. Percentages were calculated based on available data.
¶ A substantial amount of data were missing or uninformative for education.
^ Data were excluded from analysis due to limited variation.
⁺ Log_n scores in normal text and original scale scores in italics (analyses used log_n scores).
