# Supplementary material for: Long-term psychosocial outcomes of low-dose CT screening: results of the UK Lung Cancer Screening randomised controlled trial
Source: Thorax. 2016 Jul 28;71(11):996–1005. doi: 10.1136/thoraxjnl-2016-208283 (PMC5099188; doi:10.1136/thoraxjnl-2016-208283)
Supplement: Supplementary table [file thoraxjnl-2016-208283supp_table2.pdf]

Supplementary Table II. T_2 sample baseline characteristics by trial allocation

		Intervention (n=1553)[‡]<br>n (%) or mean (SD)	Control (n=1302)[‡]<br>n (%) or mean (SD)	Test statistic (p value)
**Site**	Liverpool	735 (47)	601 (46)	p=0.53
	Cambridge	818 (53)	701 (54)	
**Age (years)**		67.77 (3.98)	67.82 (3.98)	p=0.77
**Gender**	Male	1189 (77)	977 (75)	p=0.34
	Female	364 (23)	325 (25)	
**Education**[¶]	Up to GCSE/O level	509 (44)	431 (44)	p=0.77
	Beyond GCSE/O level	640 (56)	556 (56)	
**Ethnicity**	White	1536 (99)	1289 (99)	^
	Non-white	11 (1)	10 (1)	
**Marital group**	Married /cohabiting	1171 (76)	1003 (77)	p=0.37
	Not married/cohabiting	377 (24)	298 (23)	
**IMD**	Quintile 1 (most deprived)	378 (24)	290 (22)	p=0.63
	Quintile 2	186 (12)	152 (12)	
	Quintile 3	287 (19)	236 (18)	
	Quintile 4	286 (18)	259 (20)	
	Quintile 5 (least deprived)	416 (27)	365 (28)	
**Smoking status**	Current smoker	568 (37)	441 (34)	p= 0.13
	Ex-smoker	984 (63)	861 (66)	
	Never smoker	1 (<1)	0 (0)	^
**Experience of lung cancer (T_0)**	No	916 (59)	767 (59)	p=0.98
	Yes	634 (41)	532 (41)	
**Cancer distress (T0)**[+]		2.16 (.28)<br>*8.69*	2.15 (.28)<br>*8.58*	p=0.22
**Anxiety (T_0)**[+]		1.52 (.71)<br>*3.56*	1.50 (.71)<br>*3.47*	p=0.46
**Depression (T_0)**[+]		1.26 (.67)<br>*2.54*	1.25 (.66)<br>*2.47*	p=0.48
**Decision satisfaction (T_0)**	Not very satisfied	932 (60)	736 (57)	p=0.07
	Very satisfied	618 (40)	561 (43)	

‡ Ns vary in each cell due to missing data. Percentages were calculated based on available data.

¶ A substantial amount of data were missing or uninformative for education.

^ Data were excluded from analysis due to limited variation.

+ Log$_n$ scores in normal text and original scale scores in italics (analyses used log$_n$ scores).
